# Does Corporate Social Responsibility Influence Customer Loyalty? Insights from the Hotel Industry

**DOI:** 10.12688/f1000research.169211.1

**Published:** 2025-09-22

**Authors:** Ajit Kumar Singh, Saurabh Bharti, Ajay Kumar Poddar, Sandeep Paatlan, Amit Kumar Dashora, Shobhna Poddar, Mohit Dahiya, Anita Kumari Singh

**Affiliations:** 1 Chandigarh University, Sahibzada Ajit Singh Nagar, Punjab, India; 2 Welcomgroup Graduate School of Hotel Administration, Manipal Academy of Higher Education, Manipal, Karnataka, India; 3 Texila American University, Lusaka, Zambia; 4 Galgotias University, Greater Noida, Uttar Pradesh, India; 5 University of Edenberg, Lusaka, Zambia; 6 University of Sunderland, Sunderland, London, UK

**Keywords:** hotel reputation, customer satisfaction, corporate social responsibility, customer loyalty

## Abstract

**Background:**

This study investigates the relationship between customers’ perceptions of corporate social responsibility (CSR), hotel reputation, and customer loyalty within the hospitality sector. This study explored how customers’ evaluations of corporate social responsibility (CSR) initiatives influence their loyalty behaviors and whether this relationship is mediated by the hotel’s perceived reputation. This study contributes to the literature by integrating corporate social responsibility and hotel reputation into a unified model to predict customer loyalty in the hospitality sector.

**Methods:**

Data were collected through a structured questionnaire administered via convenience sampling, resulting in 391 valid responses from customers who stayed in star-rated hotels in New Delhi, India. The proposed hypotheses were assessed using PLS-SEM, and the conceptual model was further evaluated for its explanatory and predictive power.

**Results:**

The study revealed that corporate social responsibility and hotel reputation significantly and positively impact customer loyalty. Furthermore, hotel reputation partially mediates the relationship between corporate social responsibility and customer loyalty. The model demonstrated good explanatory power (R ^2^ = 0.435 for customer loyalty) and medium predictive relevance (Q ^2^ > 0.15), supporting the robustness of the proposed structural framework.

**Conclusions:**

The findings of this study reveal that corporate social responsibility significantly enhances customer loyalty. The partial mediating effect of hotel reputation suggests that while corporate social responsibility independently influences customer loyalty, its impact is further strengthened when accompanied by a strong hotel reputation. This study highlights the strategic importance of aligning corporate social responsibility initiatives with reputation-building efforts to foster deeper emotional and behavioral loyalty among customers.

## 1. Introduction

In the hospitality sector, CSR has emerged significantly and gained a competitive edge, affecting not only direct customer assessments but also intangible elements, such as a hotel’s reputation. As this sector heavily relies on building its image ( Agu et al., 2024), CSR is instrumental in sustaining hotel reputation. Many hotels are aligning their CSR initiatives with Sustainable Development Goals (SDGs) to address broader global challenges. Corporate Social Responsibility (CSR) practices significantly impact different business verticals and corporate reputation ( Su et al., 2014). Several studies in different sectors have demonstrated that CSR influences customer loyalty (CSR – CL) through mediators such as customer satisfaction and trust ( Lee, 2018; Leclercq-Machado et al., 2022; Al-Ghamdi & Badawi, 2019; Chung et al., 2015; Park & Kim, 2018). Although many studies have advocated the significance of CSR across various sectors, much of the work remains either theoretical or focused on Western or developed nations, with limited empirical investigation into the Indian hospitality industry ( Fatma et al., 2015). In India, where consumer expectations are evolving due to awareness of sustainability and ethical consumption, empirical studies on CSR and customer behavior are scarce. The role of hotel reputation as a mediator has not been thoroughly examined in this context. Hence, the present study seeks to overcome this gap by analyzing empirical data from star-rated hotel guests in New Delhi, India, and investigating both the explanatory and predictive capabilities of the suggested theoretical framework.

## 2. Literature review and hypothesis development

### 2.1 CSR and customer loyalty

Hotels classify CSR practices into many verticals, such as philanthropic, legal-reactive, and active approaches, with the primary objective of enhancing economic performance. This classification helps us understand the CSR management model that benefits the industry ( Peña-Miranda et al., 2021). Many organizations have integrated CSR into their operational strategies and promoted company sustainability and stakeholder engagement ( Bohdanowicz & Zientara, 2008). However, CSR activities related to community engagement and environmental issues do not always significantly impact reputation, indicating that integrating CSR with organizational culture is crucial for performance outcomes ( González-Rodríguez et al., 2019). Recent shifts towards communication and reporting suggest a growing emphasis on transparency and accountability in CSR practices ( Moyeen & Mehjabeen, 2024). CSR initiatives have been widely recognized as tools for enhancing business performance and stakeholder perceptions ( Sultan et al., 2024). Many studies confirm that CSR positively influences customer loyalty through different mediating factors such as image, trust, customer satisfaction, and social platform use ( Liu et al., 2019; Su et al., 2014; Mohammed & Al-Swidi, 2019; Islam et al., 2022). The above arguments support the development of H1 i.e,

H1
*Customer perception significantly affects Customer loyalty.*

### 2.2 CSR and hotel reputation

CSR not only affects hotel reputation but also customers’ emotions ( Su et al., 2014). A positive hotel reputation further impacts customer loyalty ( Kandampully & Suhartanto, 2000). For hotels, it is essential to comprehend the link between customer satisfaction and their intention to return ( Arora & Singer, 2006). Furthermore, the quality of service provided by a hotel is equally significant, as it greatly influences customer satisfaction and loyalty ( Akbar et al., 2010). CSR initiatives enhance a hotel’s reputation, which in turn boosts trust, satisfaction, and loyalty towards the company.

Such initiatives further build customer satisfaction, which serves as a mediator between CSR and CL ( Lee, 2018). Additionally, a hotel’s ethical perception can enhance customer identification with the brand, leading to emotional commitment and brand trust, thus ensuring long-term loyalty ( Fatma & Rahman, 2017). Moreover, customer experiences in full-service hotels, which encompass functional, emotional, and social dimensions, significantly impact brand trust, leading to CL ( Guan et al., 2021). The dynamic interplay between quality of service, hotel image, and customer satisfaction plays a fundamental role in establishing customer loyalty. Each of these elements contributes uniquely yet interdependently to shaping consumers’ overall perception of a brand or organization. High service quality enhances perceived value, and a strong corporate image fosters trust and credibility. Collectively, they influence consumer attitudes and behaviors, thereby serving as essential drivers in the creation and maintenance of a stable, committed, and loyal customer base ( Cheng, 2014). The above arguments advocate the development of H2, that is

H2
*Customer perception of CSR significantly affects hotel reputation.*

### 2.3 Mediating effect of hotel reputation in the CSR – CL linkage

CSR activities contribute not only to social value but also to strengthening hotel reputation and enhancing customer loyalty. It positively affects hotel reputation, which subsequently increases customer loyalty through improved trust. Reputation is a trust-building factor that deals with intangible services. However, limited research has explored how reputation acts as a psychological factor that transforms CSR perceptions into loyalty behavior ( He & Li, 2011; Martínez & Rodríguez del Bosque, 2013). Stakeholder theory suggests that customers tend to reward companies that reflect their social values, although these rewards are typically not immediate and instead manifest through constructs such as reputation, which accumulate perceptions over time ( Fombrun & Shanley, 1990; Freeman, 1984). When hotels implement CSR activities, such as promoting environmental sustainability, engaging with the community, or adopting ethical labor practices, they send positive messages to the public, contributing to the development of a strong, positive corporate reputation ( He & Li, 2011). This reputation, in turn, enhances customer trust, brand admiration, and psychological closeness, all of which are precursors to loyalty behaviors such as repeat visits and advocacy ( Walsh, Beatty, & Shiu, 2009). CSR activities have significantly boosted the public image of hotels, and this enhanced reputation directly influences customers’ willingness to recommend and return ( Lee et al., 2013). This suggests that hotel reputation works as a value transformation mechanism, where a brand’s ethical and social efforts are converted into loyalty outcomes through positive stakeholder perception. Kang, Lee, and Huh (2010) also highlighted that while CSR directly impacts performance, its effect is magnified when the hotel is seen as reputable and credible. In other words, CSR alone may not be sufficient to secure customer loyalty unless customers perceive it as a reputation-enhancing activity ( Pérez & Rodríguez del Bosque, 2015). Customers often cannot directly assess the authenticity of CSR actions; therefore, reputation serves as a cognitive shortcut that helps them infer the long-term intentions and reliability of the hotel brand ( Groza et al., 2011). When both CSR and reputation are viewed positively, customers are more likely to develop affective commitments, making them less sensitive to price and more emotionally invested in the brand. Thus, hotel reputation is not just a by-product of CSR but a strategic asset that mediates trust-building and relational value derived from socially responsible practices. Thus, the arguments presented above provide a basis for formulating H3 and H4, respectively.

H3
*Hotel reputation significantly affects customer loyalty.*

H4
*Hotel reputation mediates the relationship between CSR and customer loyalty.*

**Figure 1.**
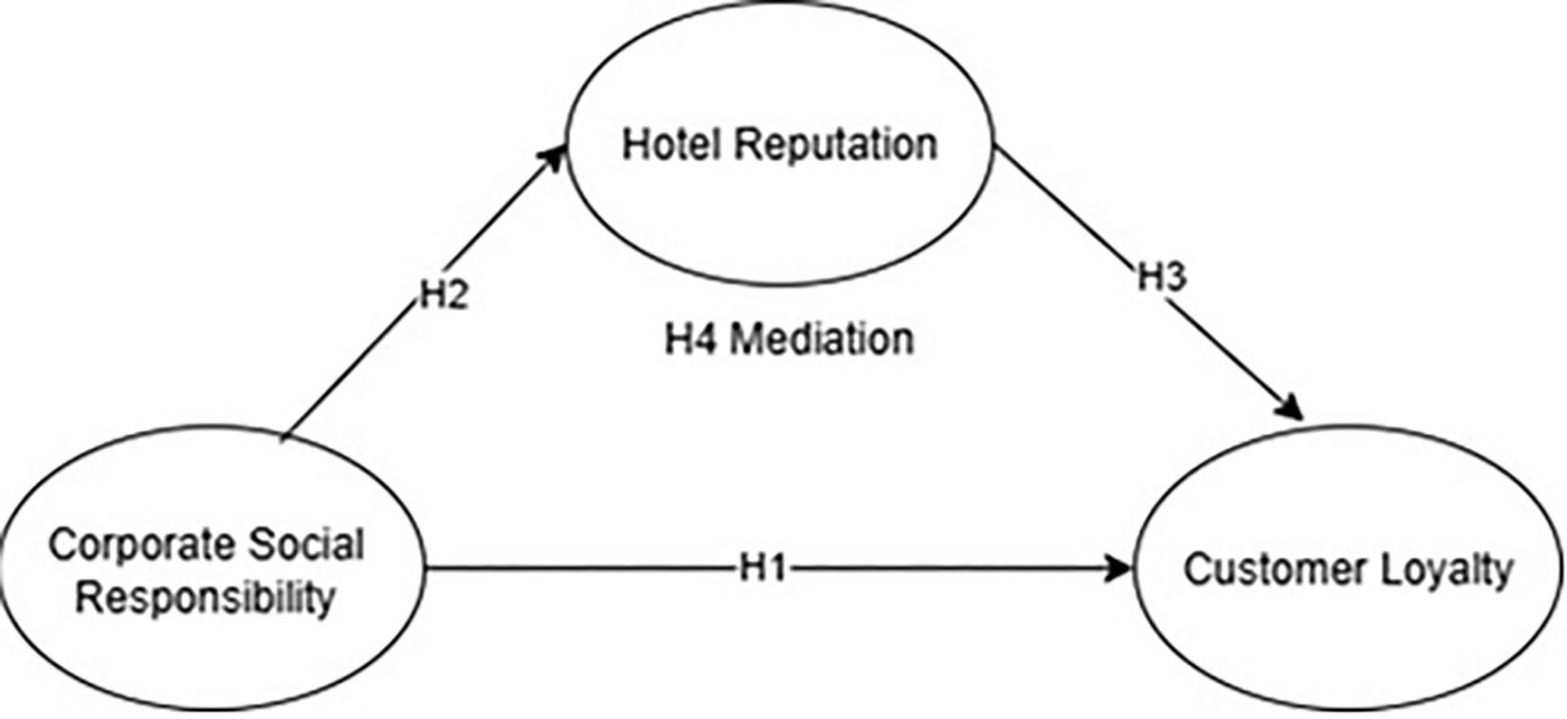
Author’s proposed model.

The conceptual framework for the present study is depicted in Figure 1.

To empirically examine the hypothesized relationships among the constructs CSR, HR, and CL, a system of linear equations is proposed.

$$\mathrm{HR}=\beta_{1}\cdot \mathrm{CSR}+\varepsilon_{1}$$ (1)

$$\mathrm{CL}=\beta_{2}\cdot \mathrm{CSR}+\beta_{3}\cdot \mathrm{HR}+\varepsilon_{2}$$ (2)

Whereas:

• CSR = Customer perception of corporate social responsibility
• HR = Hotel reputation (mediator)
• CL = Customer loyalty (dependent variable)
• β _1_, β _2_, β _3_ = Path coefficients to be estimated
• ε _1_, ε _2_ = Error terms

The first equation expresses HR as a function of CSR. This implies that hotel’s CSR has a direct influence on how the hotel’s reputation is perceived, where β _1_ represents the path coefficient, and **ε _1_** accounts for unexplained variance or error. The second equation outlines CL as influenced by both CSR and HR. Here, CL is shaped not only directly by CSR (coefficient β _2_) but also indirectly through the mediating effect of Hotel Reputation (coefficient β _3_). **ε _2_** represents the residual error in predicting customer loyalty.

## 3. Research methodology

**Figure 2.**
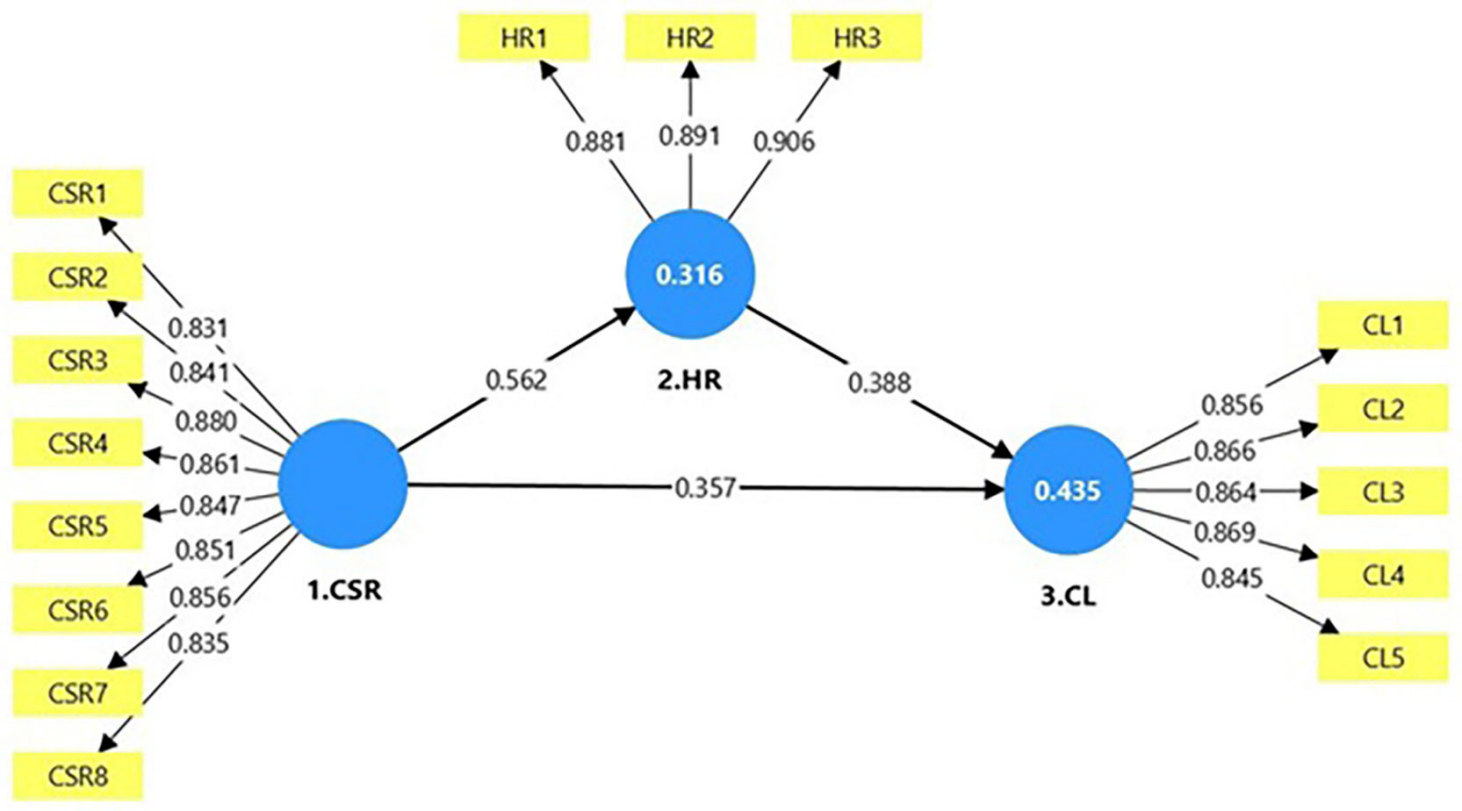
Measurement model illustration using PLS Algorithm. Source: Developed by the author.

This study uses cross-sectional data, and partial least squares structural equation Modelling is employed for model validation and hypothesis testing ( Figure 2). PLS-SEM does not assume multivariate normality and demonstrates robustness when applied to small sample sizes ( Hair et al., 2016). For data collection, a convenience sampling method was used. Structured questionnaires were distributed to customers staying at star-rated hotels in New Delhi, India, between August 5 and 9, 2025. The questionnaire used in this study was developed by the authors and comprised items adapted from previously validated research instruments. All participants in this study were aged 18 years or more. Of the 425 questionnaires distributed, 34 were excluded, and 391 were considered for the study. The items included in the questionnaire were evaluated using a five-point Likert scale, which allowed respondents to indicate their level of agreement with each statement. The scale ranged from 1 to 5, where 1 corresponded to ‘strongly disagree’ and 5 represented ‘strongly agree.’ The CSR construct was evaluated using eight items ( El Akremi et al., 2018; Kim & Kim, 2016), Customer Loyalty (CL) with five items derived from Zeithaml et al. (1996), and Hotel Reputation (HR) through three items ( Chun, 2005). Table 1 presents the descriptive statistics of the variables.

**Table 1.** Descriptive statistics.

Measured Items	Mean	Standard deviation	Kurtosis	Skewness
CSR1	2.601	0.611	0.036	0.15
CSR2	2.949	0.609	0.645	0.163
CSR3	2.706	0.614	0.079	0.076
CSR4	2.691	0.655	0.12	-0.017
CSR5	2.831	0.629	0.359	0.085
CSR6	3.013	0.642	0.519	0.222
CSR7	2.969	0.659	0.301	0.033
CSR8	2.992	0.661	0.108	0.062
HR1	2.99	0.75	0.153	0.017
HR2	3.031	0.796	-0.017	-0.116
HR3	2.724	0.803	0.083	0.155
CL1	2.954	0.696	0.518	0.017
CL2	2.977	0.772	-0.093	0.04
CL3	3.422	0.707	0.217	-0.071
CL4	3.018	0.752	0.127	0.043
CL5	2.926	0.745	-0.124	0.009

Source: Compiled by the author.

### 3.1 Measurement model

**Figure 3.**
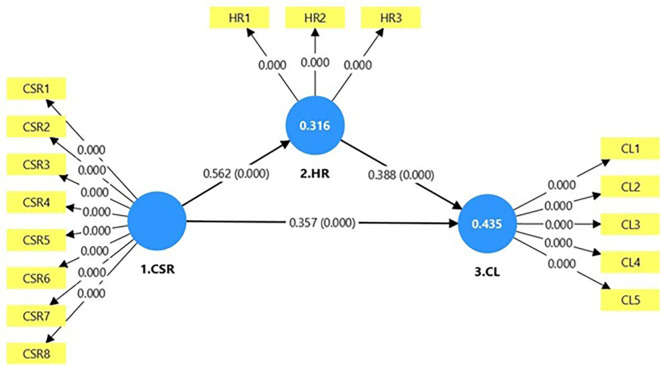
Structural model illustration using Bootstrapping. Source: Developed by the author.

Figure 3 illustrates the outer and inner loadings of the measurement model. To assess the internal consistency of the constructs, multiple reliability indicators were employed, including Cronbach’s alpha, rho_A, and composite reliability (rho_ C) ( Table 2). The results revealed that all factor loadings were above the acceptable threshold of 0.70, indicating strong reliability. Furthermore, Cronbach’s alpha values exceeded 0.70 for all constructs, indicating acceptable internal consistency. The rho_A values, which provide a more accurate estimate of construct reliability in certain contexts, fell between Cronbach’s alpha and rho_ C values and consistently remained above 0.70. This alignment among the reliability measures confirms that the constructs exhibit strong internal reliability and consistency. To establish convergent validity, the average variance extract was used. As presented in Table 2, the Average Variance Extracted (AVE) values for all constructs were greater than the recommended threshold of 0.50. This indicates that the measurement model exhibited acceptable convergent validity, confirming that the items intended to measure each construct shared a sufficient proportion of common variance. In addition to convergent validity, discriminant validity was assessed using several approaches. These included examining the cross-loadings of each indicator, applying the Fornell–Larcker criterion, and calculating the hetero trait–mono trait ratio of correlations (HTMT). The results from all three methods demonstrated that the constructs CSR, HR, and CL are empirically distinct from one another, thereby supporting the establishment of discriminant validity. The outcomes of all three tests are detailed in Table 3, Table 4, and Table 5. Multicollinearity for all factors was examined using VIF ( Table 2). The VIF values for all factors were below 5, suggesting no multicollinearity concerns.

**Table 2.** Factor loadings, reliability and validity.

	Factor Loadings	Cronbach’s alpha	(rho_A)	(rho_ C)	(AVE)	VIF
Corporate Social Responsibility		0.945	0.946	0.954	0.723	
CSR1	0.831					2.542
CSR2	0.841					2.741
CSR3	0.880					3.501
CSR4	0.861					3.095
CSR5	0.847					2.84
CSR6	0.851					2.938
CSR7	0.856					2.892
CSR8	0.835					2.648
Hotel Reputation		0.873	0.877	0.922	0.797	
HR1	0.881					2.25
HR2	0.891					2.337
HR3	0.906					2.425
Customer Loyalty		0.912	0.913	0.934	0.740	
CL1	0.856					2.513
CL2	0.866					2.618
CL3	0.864					2.555
CL4	0.869					2.646
CL5	0.845					2.356

Source: Compiled by the author.

**Table 3.** Discriminant validity - HTMT –test.

	CSR	HR	CL
CSR			
HR	0.617		
CL	0.619	0.658	

Source: Compiled by the author.

**Table 4.** Discriminant validity - Fornell & Larcker – test.

	CSR	HR	CL
CSR	**0.85**		
HR	0.562	**0.893**	
CL	0.576	0.589	**0.86**

Source: Compiled by the author.

**Table 5.** Discriminant validity–cross loadings.

	CSR	HR	CL
CSR1	0.831	0.506	0.488
CSR2	0.841	0.477	0.455
CSR3	0.880	0.502	0.526
CSR4	0.861	0.461	0.510
CSR5	0.847	0.465	0.484
CSR6	0.851	0.467	0.474
CSR7	0.856	0.496	0.492
CSR8	0.835	0.447	0.484
HR1	0.482	0.881	0.485
HR2	0.492	0.891	0.515
HR3	0.529	0.906	0.573
CL1	0.495	0.483	0.856
CL2	0.489	0.521	0.866
CL3	0.511	0.529	0.864
CL4	0.503	0.507	0.869
CL5	0.478	0.493	0.845

Source: Compiled by the author.

### 3.2 Structural model

The hypothesized relationships among the three core constructs–CSR, HR, and CL–are shown in Figure 3. The model was tested using path analysis with the PLS-SEM approach.

H1 explores the link between CSR and CL (CSR → CL). The analysis reveals that CSR significantly impacts CL (β = 0.357, t = 8.456, p < 0.01), thereby validating H1. H2 considers whether CSR is significantly linked to HR (CSR → HR). The findings confirm that CSR significantly impacts HR (β = 0.562, t = 16.104, p < 0.01), confirming H2. H3 investigates the significant association between HR and CL (HR → CL). The results showed that HR significantly impacted CL (β = 0.388, t = 8.823, p < 0.01), leading to the acceptance of H3. The outcomes are presented in Table 6.

**Table 6.** Hypothesis testing.

	β	SD	t- values	p-values	Result
H1: CSR -> CL	0.357	0.042	8.456	0.000	Accepted
H2: CSR -> HR	0.562	0.035	16.104	0.000	Accepted
H3: HR -> CL	0.388	0.044	8.823	0.000	Accepted

Source: Author’s own work.

### 3.3 Mediation analysis

H4 examined whether HR acts as a mediator between CSR and CL. Specifically, it examined whether the influence of CSR on CL is transmitted through HR, forming an indirect path (CSR → HR → CL). The results revealed a statistically significant indirect effect (β = 0.218, t = 7.807, p < 0.05). This indicates that CSR positively influences CL through its effect on HR. Path analysis showed that in the presence of HR as a mediator, the direct effect of CSR on CL remained statistically significant (β = 0.357, t = 8.456, p < 0.01). As both effects were significant, this suggests that HR partially mediates the CSR-CL relationship ( Table 7).

**Table 7.** Mediation analysis.

Total effect		Direct effect		Specific indirect effect						
β	p-value	B	P-value	H9: CSR -> HR -> CL	β	t-value	Upper limit	Lower limit	p-value	Results
0.576	0.000	0.357	0.000		0.218	7.807	0.174	0.267	0.000	Partial Mediation

Source: Author’s own work.

Table 8 presents the findings related to the model’s explanatory and predictive strengths. The R ^2^ value for CL was 0.435, indicating that the combined effects of CSR and Hotel Reputation (HR) accounted for 43.5% of the variance in CL. The results show a moderate level of explanatory power. Hence, the model effectively captures the key determinants of customer loyalty in the hotel industry.

**Table 8.** Model’s explanatory and predictive power.

Predictor(s)	Endogenous variable	R ^2^	f ^2^	Q ^2^
CSR	CL	0.435	0.154	0.327
HR			0.182	
CSR	HR	0.316	0.462	0.313

Source: Author’s own work.

The results also revealed that both CSR and HR exert a moderate effect on CL (f ^2^ > 0.15), while CSR alone demonstrated a large effect size (f ^2^ > 0.35), underscoring its substantial role in shaping customer loyalty. The Q ^2^ value was assessed using a blindfolding procedure. The Q ^2^ values for HR and CSR were 0.327 and 0.313, respectively, both surpassing the recommended threshold of 0.15, as suggested by Hair et al. (2013). Hence, the model possesses medium predictive relevance, indicating its capability to predict endogenous constructs with reasonable accuracy.

## 4. Discussion and conclusion

The study reveals a significant association between CSR and CL and investigates both direct and indirect links among CSR, HR, and CL, contributing to both theoretical understanding and practical applications. The direct and positive relationship between CSR-CL supports Hypothesis 1 ( Lee et al., 2013; Kim & Kim, 2016). This suggests that customers significantly consider hotels’ CSR efforts when developing their emotional and behavioral loyalty. Hypothesis 2 is also validated, demonstrating CSR’s significant effect on HR. These findings underscore CSR’s function as a reputational signal, influencing customer perceptions of trustworthiness, quality, and credibility ( Chun, 2005). Furthermore, HR significantly impacts customer loyalty, confirming Hypothesis 3. This connection illustrates the mediating process through which CSR boosts loyalty not only by directly engaging customers but also by enhancing the hotel’s perceived market reputation. The path analysis in this study indicates that in the presence of the HR mediator, the direct impact of CSR on CL remains significant. As both the direct and specific indirect effects were significant, HR partially mediated the CSR-CL relationship, confirming Hypothesis 4. The partial mediation observed implies that while CSR independently influences loyalty, a substantial portion of its effect is mediated by the improvement of hotel reputation. This implies that reputation serves as a conduit for translating CSR initiatives into customer loyalty. From a model evaluation perspective, the R ^2^ value of 0.435 for customer loyalty demonstrates a moderate explanatory power. Moreover, the effect size analysis indicates that CSR has a large effect (f ^2^ > 0.35) on CL, while both CSR and HR exert moderate effects. The Q ^2^ values for HR (0.327) and CSR (0.313) confirm the medium predictive relevance of the model, reinforcing its validity for understanding and forecasting customer behavior in the luxury hotel segment. The findings of this study emphasize the need for hospitality firms to strategically oversee their CSR endeavors and reputation-building strategies, as both are crucial for enhancing customer loyalty. Furthermore, this study sets the stage for future investigations that might examine other possible mediators or moderators, such as customer trust or perceived service quality, to further elucidate CSR–loyalty linkages. These findings are particularly significant in the post-pandemic context, where consumer expectations regarding ethical and responsible business practices have intensified. Hotels that invest in socially responsible activities not only differentiate themselves in a competitive market but also cultivate deeper emotional and attitudinal bonds with their clients.

## 5. Practical implications

CSR in the hotel industry goes beyond simple philanthropy ( Lee et al., 2013). Customers often view these efforts as indicators of a hotel’s overall integrity and service quality, thereby strengthening their loyalty intentions ( Han, Yu, & Kim, 2018). This study presents several key practical implications for the hospitality sector. First, it demonstrates that customers’ perception of CSR efforts greatly affects their loyalty, both directly and indirectly, via the hotel’s reputation ( Su et al., 2014). This shows that socially responsible practices are fundamental to business operations, not just compliance or charitable acts, but also tools for customer-focused brand positioning. Second, hotel reputation highlights the importance of communicating CSR efforts to improve stakeholders’ views of the brand ( Lii & Lee, 2011). Hotel managers should focus on transparent communication methods, such as issuing sustainability reports, sharing stories on social media, and updating the community on engagement activities to establish and uphold a strong, credible reputation. A well-respected reputation serves as a mechanism for building trust, strengthening customers’ emotional ties to the brand, and encouraging long-term loyalty. Furthermore, this study establishes a framework for hospitality businesses to measure the return on investment (ROI) of their CSR programs, evaluating not only the social impact but also customer-centric outcomes such as loyalty and advocacy. By aligning CSR initiatives with customer values and expectations, such as environmental responsibility, employee care, and ethical sourcing, hotels can foster customer loyalty and differentiate their brand in a competitive landscape ( Islam et al., 2022). Hotel industry leaders must equip their frontline and customer-facing teams with the skills to embody and articulate their hotel’s CSR commitments. Customer experiences are shaped not only by the quality of amenities and services but also by the ethical behavior that guests perceive. Operationally, hotel leaders are encouraged to train and empower their frontline employees to internalize and communicate CSR values. Customer experiences are shaped not only by tangible services and amenities but also by ethical conduct and value alignment perceived during service encounters. Therefore, it is crucial to align internal culture with the external CSR narrative to fortify the link between CSR-HR-CL ( Osakwe & Yusuf, 2020).

## 6. Limitations and suggestions for future research

A cross-sectional design was used in this study; hence, a longitudinal research design is recommended for future research to better understand the evolving nature of CSR initiatives and their influence on customer perceptions over time. The use of convenience sampling from a single geographic location (New Delhi, India) may limit the generalizability of our findings. Researchers should adopt probability sampling across diverse geographic regions and hotel categories. Incorporating alternative mediating variables could provide deeper insights into CSR - customer loyalty research. Moreover, hotel reputation as a moderating factor could be a focus of future investigations. Comparative analyses across different tiers of hospitality establishments and regional contexts may reveal context-specific CSR strategies and their effectiveness. Future investigations should integrate internal stakeholder perspectives to examine how internal CSR engagement contributes to external brand image and guest satisfaction. Additionally, interdisciplinary research integrating psychological constructs, such as customer empathy, moral identity, or ethical consumption patterns, could further enrich the theoretical understanding of why and how CSR influences customer behavior in the hotel industry.

## Ethics statement

This study complied with the ethical guidelines of the Declaration of Helsinki and was reviewed and approved by the Ethics Committee of Texila American University, Zambia (Approval No. TAUZ/REC/2025/F/04, dated 5 August 2025). All participants were over 18 years of age and provided written informed consent prior to the data collection. The confidentiality and anonymity of the participants’ data were strictly maintained.

## Data Availability

The dataset included the values used for statistical analyses, tables, and figures, and comprised responses from 391 participants who took part in the study. The dataset is openly available on Zenodo at https://doi.org/10.5281/zenodo.15824958 ( Singh, 2025a). The questionnaire used in this study is available on Zenodo at https://doi.org/10.5281/zenodo.16220978 ( Singh, 2025b). Data are available under the terms of the Creative Commons Attribution 4.0 International license (CC-BY 4.0) https://creativecommons.org/licenses/by/4.0/.

## References

[ref1] Agu E Iyelolu T Idemudia C : Exploring the relationship between sustainable business practices and increased brand loyalty. *Int. J. Man & Ent. Res.* 2024;6(8):2463–2475. 10.51594/ijmer.v6i8.1365

[ref2] Akbar S Mat Som AP Jamil Alzaidiyeen N : Revitalization of Service Quality to Gain Customer Satisfaction and Loyalty. *Int. J. Bus. Manag.* 2010;5(6). 10.5539/ijbm.v5n6p113

[ref3] Al-Ghamdi SAA Badawi NS : Do corporate social responsibility activities enhance customer satisfaction and customer loyalty? Evidence from the Saudi banking sector. *Cogent Bus Manag.* 2019;6(1). 10.1080/23311975.2019.1662932

[ref4] Arora R Singer J : Customer Satisfaction and Value as Drivers of Business Success for Fine Dining Restaurants. *Serv. Mark. Q.* 2006;28(1):89–102. 10.1300/j396v28n01_05

[ref5] Bohdanowicz P Zientara P : Corporate Social Responsibility in Hospitality: Issues and Implications. A Case Study of Scandic. *Scand. J. Hosp. Tour.* 2008;8(4):271–293. 10.1080/15022250802504814

[ref6] Cheng B-L : Service Quality and the Mediating Effect of Corporate Image on the Relationship between Customer Satisfaction and Customer Loyalty in the Malaysian Hotel Industry. *Gadjah Mada Int. J. Bus.* 2014;15(2):99. 10.22146/gamaijb.5474

[ref7] Chun R : Corporate reputation: Meaning and measurement. *Int. J. Manag. Rev.* 2005;7(2):91–109. 10.1111/j.1468-2370.2005.00109.x

[ref8] Chung K-H Yu J-E Choi M-G : The Effects of CSR on Customer Satisfaction and Loyalty in China: The Moderating Role of Corporate Image. *J. Econ. Bus. Manag.* 2015;3(5):542–547. 10.7763/joebm.2015.v3.243

[ref9] El Akremi A Gond J-P Swaen V : How do employees perceive corporate responsibility? Development and validation of a multidimensional corporate stakeholder responsibility scale. *J. Manag.* 2018;44(2):619–657. 10.1177/0149206315569311

[ref10] Fatma M Rahman Z : An Integrated Framework to Understand How Consumer-Perceived Ethicality Influences Consumer Hotel Brand Loyalty. *Serv. Sci.* 2017;9(2):136–146. 10.1287/serv.2016.0166

[ref11] Fatma M Rahman Z Khan I : The role of CSR as a determinant of consumer responses in the financial sector. *Decision.* 2015;42(4):393–401. 10.1007/s40622-015-0091-6

[ref12] Fombrun CJ Shanley M : What’s in a name? Reputation building and corporate strategy. *Acad. Manag. J.* 1990;33(2):233–258. 10.5465/256324

[ref13] Freeman RE : *Strategic management: A stakeholder approach.* Pitman Publishing;1984.

[ref14] González-Rodríguez MR Martín-Samper RC Köseoglu MA : Hotels’ corporate social responsibility practices, organizational culture, firm reputation, and performance. *J. Sustain. Tour.* 2019;27(3):398–419. 10.1080/09669582.2019.1585441

[ref15] Groza MD Pronschinske MR Walker M : Perceived organizational motives and consumer responses to proactive and reactive CSR. *J. Bus. Ethics.* 2011;102(4):639–652. 10.1007/s10551-011-0834-9

[ref16] Guan J Wang W Chan JH : Customer experience and brand loyalty in the full-service hotel sector: the role of brand affect. *Int. J. Contemp. Hosp. Manag.* 2021;33(5):1620–1645. 10.1108/ijchm-10-2020-1177

[ref17] Hair JF Hult GTM Ringle CM : *A primer on partial least squares structural equation modeling (PLS-SEM).* SAGE Publications; 2nd ed 2016.

[ref18] Hair JF Ringle CM Sarstedt M : Partial least squares structural equation modeling: Rigorous applications, better results and higher acceptance. *Long Range Plan.* 2013;46(1–2):1–12. 10.1016/j.lrp.2013.01.001

[ref19] Han H Yu J Kim W : Environmental corporate social responsibility and the strategy to boost the airline’s image and customer loyalty intentions. *J. Air Transp. Manag.* 2018;66:20–30. 10.1016/j.jairtraman.2017.09.013

[ref20] He H Li Y : CSR and service brand: The mediating effect of brand identification and moderating effect of service quality. *J. Bus. Ethics.* 2011;100(4):673–688. 10.1007/s10551-010-0703-y

[ref21] Islam T Khan M Ghaffar A : Does CSR influence sustained competitive advantage and behavioral outcomes? An empirical study in the hospitality sector. *J. Glob. Scholars Market. Sci.* 2022;33(1):107–132. 10.1080/21639159.2022.2098157

[ref22] Kandampully J Suhartanto D : Customer loyalty in the hotel industry: the role of customer satisfaction and image. *Int. J. Contemp. Hosp. Manag.* 2000;12(6):346–351. 10.1108/09596110010342559

[ref23] Kang KH Lee S Huh C : Impacts of positive and negative corporate social responsibility activities on company performance in the hospitality industry. *Int. J. Hosp. Manag.* 2010;29(1):72–82. 10.1016/j.ijhm.2009.05.006

[ref24] Kim S Kim SY : The effect of CSR fit and CSR authenticity on the brand attitude. *Sustainability.* 2016;8(4):381. 10.3390/su8040381

[ref25] Leclercq-Machado L Alvarez-Risco A Almanza-Cruz C : Effect of Corporate Social Responsibility on Consumer Satisfaction and Consumer Loyalty of Private Banking Companies in Peru. *Sustainability.* 2022;14(15):9078. 10.3390/su14159078

[ref26] Lee C-Y : Does Corporate Social Responsibility Influence Customer Loyalty in the Taiwan Insurance Sector? The role of Corporate Image and Customer Satisfaction. *J. Promot. Manag.* 2018;25(1):43–64. 10.1080/10496491.2018.1427651

[ref27] Lee S Park SY Lee J : Raising corporate reputation through corporate social responsibility and green management: The case of the hotel industry. *Int. J. Hosp. Manag.* 2013;35:102–112. 10.1016/j.ijhm.2013.05.002

[ref28] Lii Y-S Lee M : Doing Right Leads to Doing Well: When the Type of CSR and Reputation Interact to Affect Consumer Evaluations of the Firm. *J. Bus. Ethics.* 2011;105(1):69–81. 10.1007/s10551-011-0948-0

[ref29] Liu MT Zhao Z Zhu Z : How CSR influences customer behavioural loyalty in the Chinese hotel industry. *Asia Pac. J. Mark. Logist.* 2019;32(1):1–22. 10.1108/apjml-04-2018-0160

[ref30] Martínez P Rodríguez del Bosque I : CSR and customer loyalty: The roles of trust, customer identification with the company and satisfaction. *Int. J. Hosp. Manag.* 2013;35:89–99. 10.1016/j.ijhm.2013.05.009

[ref31] Mohammed A Al-Swidi A : The influence of CSR on perceived value, social media and loyalty in the hotel industry. *Spanish Journal of Marketing - ESIC.* 2019;23(3):373–396. 10.1108/sjme-06-2019-0029

[ref32] Moyeen A Mehjabeen M : CSR research in the hotel industry: how it relates to promoting the SDGs. *Social Responsibility Journal.* 2024;20(9):1770–1786. 10.1108/srj-01-2024-0032

[ref33] Osakwe CN Yusuf TO : CSR: a roadmap towards customer loyalty. *Total Qual. Manag. Bus. Excell.* 2020;32(13–14):1424–1440. 10.1080/14783363.2020.1730174

[ref34] Park E Kim KJ : What drives “customer loyalty”? The role of corporate social responsibility. *Sustain. Dev.* 2018;27(3):304–311. 10.1002/sd.1901

[ref35] Peña-Miranda DD Fraiz-Brea JA Camilleri MA : Corporate social responsibility model for a competitive and resilient hospitality industry. *Sustain. Dev.* 2021;30(3):433–446. 10.1002/sd.2259

[ref36] Pérez A Rodríguez del Bosque I : Corporate social responsibility and customer loyalty: Exploring the role of identification, satisfaction and type of company. *J. Serv. Mark.* 2015;29(1):15–25. 10.1108/JSM-10-2013-0272

[ref37] Singh AK : Data of 391 Respondents_CSR_HR_CL.[Data set]. *Zenodo.* 2025a. 10.5281/zenodo.15824958

[ref38] Singh AK : Questionnaire.[Data set]. *Zenodo.* 2025b. 10.5281/zenodo.16220978

[ref39] Su L Huang S(S) Van Der Veen R : Corporate Social Responsibility, Corporate Reputation, Customer Emotions and Behavioral Intentions: A Structural Equation Modeling Analysis. *J. China Tour. Res.* 2014;10(4):511–529. 10.1080/19388160.2014.958606

[ref40] Sultan MF Shaikh E Tunio MN : *Strategic Impact of Corporate Social Responsibility.* igi global;2024;23–33. 10.4018/979-8-3693-0363-4.ch002

[ref41] Walsh G Beatty SE Shiu EM : The customer-based corporate reputation scale: Replication and short form. *J. Bus. Res.* 2009;62(10):924–930. 10.1016/j.jbusres.2007.11.018

[ref42] Zeithaml VA Berry LL Parasuraman A : The behavioral consequences of service quality. *J. Mark.* 1996;60(2):31–46. 10.1177/002224299606000203

