# Supplementary material for: Does Corporate Social Responsibility Influence Customer Loyalty? Insights from the Hotel Industry
Source: F1000Res. 2025 Sep 22;14:962. [Version 1] doi: 10.12688/f1000research.169211.1 (PMC12501588; doi:10.12688/f1000research.169211.1)
Supplement: Peer review history

# Reviewer report: Reviewer response for version 1

**DOI:** 10.5256/f1000research.186505.r417238

**Published:** 2025-10-08

**Authors:** Dr Oyelakin Idris Oyewale

**Affiliations:** 1 Business and Communication, INTI International University & Colleges, Nilai, Negeri Sembilan, Malaysia

**Title:**

 The current title does not fully reflect the study framework. Since *hotel reputation* is a key variable in the research, it should be explicitly included in the title to align

 with the study’s conceptual model.

**Abstract**

- The abstract contains repetition, particularly in the first and second sentences, both of which explain the study’s objective. It is advisable to retain the second sentence while revising it to include the research problem. This will establish a clear rationale and underscore the need for the study.
- The methodology section is absent. To enhance clarity and coherence, I recommend applying the **POMES framework** (Problem, Objective, Methodology, Expected Outcome, and Significance). This structure will ensure the abstract communicates the essence of the study effectively.

**Introduction**

 The introduction provides a review of literature and identifies existing gaps. However, the **research problem is not explicitly highlighted**. To strengthen the rationale and demonstrate the urgency of the study, the research problem should be clearly articulated.

**Methodology**

- The study employed a convenience sampling method, but no justification was provided for its use. A rationale should be included to demonstrate why this technique was appropriate and acceptable in the context of the research.
- The exclusion of **34 respondents** from the dataset requires explanation. Clarifying the reasons for their removal will strengthen the credibility, authenticity, and reliability of the data analysis.

**Contribution**

 The study highlights some practical contributions. However, **policy recommendations** are currently absent. Including actionable policy contributions would significantly enhance the practical relevance and impact of the study, particularly for decision-makers in the hospitality industry.

Is the work clearly and accurately presented and does it cite the current literature?

Yes

If applicable, is the statistical analysis and its interpretation appropriate?

Yes

Are all the source data underlying the results available to ensure full reproducibility?

Yes

Is the study design appropriate and is the work technically sound?

Yes

Are the conclusions drawn adequately supported by the results?

Yes

Are sufficient details of methods and analysis provided to allow replication by others?

Partly

Reviewer Expertise:

CSR

I confirm that I have read this submission and believe that I have an appropriate level of expertise to confirm that it is of an acceptable scientific standard.

# Reviewer report: Reviewer response for version 1

**DOI:** 10.5256/f1000research.186505.r417239

**Published:** 2025-10-06

**Authors:** Varinder Singh Rana

**Affiliations:** 1 City University College of Ajman, Ajman, Ajman, United Arab Emirates

This study makes a valuable contribution to the hospitality literature by exploring the link between corporate social responsibility (CSR), hotel reputation, and customer loyalty. The research is timely, well-structured, and supported by a robust methodology using PLS-SEM with an adequate sample size.

 Findings confirm that CSR and hotel reputation significantly enhance loyalty, with reputation serving as a partial mediator. The paper is clearly written, offers meaningful theoretical and practical implications, and highlights the strategic role of aligning CSR initiatives with reputation-building.

 However, the study would benefit from strengthening the literature review with recent sources and theoretical frameworks, providing more detail on measurement validity, and acknowledging sampling limitations. Addressing these points will further enhance its scientific soundness and impact.

Is the work clearly and accurately presented and does it cite the current literature?

Yes

If applicable, is the statistical analysis and its interpretation appropriate?

Yes

Are all the source data underlying the results available to ensure full reproducibility?

Yes

Is the study design appropriate and is the work technically sound?

Yes

Are the conclusions drawn adequately supported by the results?

Yes

Are sufficient details of methods and analysis provided to allow replication by others?

Yes

Reviewer Expertise:

Hospitality and Tourism, Events, Marketing

I confirm that I have read this submission and believe that I have an appropriate level of expertise to confirm that it is of an acceptable scientific standard.

# Reviewer report: Reviewer response for version 1

**DOI:** 10.5256/f1000research.186505.r417232

**Published:** 2025-09-30

**Authors:** Dr. Uma Pandey

**Affiliations:** 1 Jagran Lakecity University, India, Bhopal, India

1. At present, a few sections (e.g., Introduction, Literature Review, and some reference titles) use *“consumer.”* For clarity and alignment with your research objectives, I recommend standardizing terminology so that *“customer”* is used consistently. This will avoid confusion for readers and strengthen the focus of your argument on hotel customers rather than the broader concept of consumers.

 2. The abbreviation “CL” is used to represent Customer Loyalty throughout the paper, but it is never formally introduced at first mention. For clarity and consistency, the authors should define all abbreviations (CSR, HR, CL) when they first appear and ensure uniform usage across the text, tables, and figures.

 3. Section 2.1 (CSR and Customer Loyalty):

 The review discusses how CSR practices influence customer loyalty. However, the hypothesis (H1) is formulated around customer perception of CSR. This creates a conceptual gap, as the supporting literature largely emphasizes CSR practices rather than perceptions. To address this, I suggest either:

 (a) Adjusting the wording of H1 to align with the CSR practice-focused literature, or

 (b) Expanding the literature review with studies that specifically examine perceived CSR and its effect on loyalty.

 This adjustment would ensure the hypothesis is firmly grounded in prior research.

 4. Section 2.2 (CSR and Hotel Reputation):

 A similar issue arises with H2, where the cited studies demonstrate how CSR initiatives influence reputation, but the hypothesis is framed around customer perception of CSR. To improve coherence, the authors should either:

 (a) Reframe H2 to emphasize CSR practices, or

 (b) Incorporate literature explicitly linking perceived CSR to hotel reputation.

 In addition, clarifying early on that CSR is conceptualized in this study as customer perception would resolve potential confusion.

 5. Section 2.3 (Hotel Reputation and Customer Loyalty):

 H3 (“Hotel reputation significantly affects customer loyalty”) is not sufficiently supported in the current literature review. While the discussion addresses mediation (CSR → Reputation → Loyalty), it does not clearly establish the direct relationship between reputation and loyalty. To strengthen this section, please integrate studies that explicitly test the direct effect. This will allow for a more logical sequence: (i) establish the direct effect (H3) and then (ii) build the case for the mediation hypothesis (H4).

 6. The discussion overemphasizes CSR outcomes and should clarify why customers value CSR, while distinguishing between attitudinal and behavioral loyalty to match the measurement model. The conclusion repeats prior points and could be condensed into clearer managerial takeaways, highlighting that CSR is framed as customer perception. Practical implications are relevant but too general; more actionable guidance (e.g., staff training, feedback-based CSR evaluation, aligning CSR initiatives with customer values) would strengthen the paper.

 7. The study would benefit from recent citations from 2024 and 2025.

 8. The title can be reframed to include the mediating role of hotel reputation.

Is the work clearly and accurately presented and does it cite the current literature?

Partly

If applicable, is the statistical analysis and its interpretation appropriate?

I cannot comment. A qualified statistician is required.

Are all the source data underlying the results available to ensure full reproducibility?

Yes

Is the study design appropriate and is the work technically sound?

Yes

Are the conclusions drawn adequately supported by the results?

Partly

Are sufficient details of methods and analysis provided to allow replication by others?

Yes

Reviewer Expertise:

Tourism, Hospitality, Aviation, Gender Studies, Marketing, AI in Tourism

I confirm that I have read this submission and believe that I have an appropriate level of expertise to confirm that it is of an acceptable scientific standard, however I have significant reservations, as outlined above.

# Reviewer report: Reviewer response for version 1

**DOI:** 10.5256/f1000research.186505.r417231

**Published:** 2025-09-30

**Authors:** Dr Priyakrushna Mohanty

**Affiliations:** 1 Christ University, Bengaluru, India

I have gone through the paper thoroughly and I am pretty much impressed with the work. There are, however, some statements that might require explanation or theoretical backing. This is not a mandate, but may be required for better positioning the paper.

 1. The use of convenience sampling for the study needs to be justified.

 2.  Hotel reputation measured with only three items which may be difficult to operationalize. Authors may explain as to why there are only 3 measures selected.

 Other than that, everything looks fine.

Is the work clearly and accurately presented and does it cite the current literature?

Yes

If applicable, is the statistical analysis and its interpretation appropriate?

Yes

Are all the source data underlying the results available to ensure full reproducibility?

Yes

Is the study design appropriate and is the work technically sound?

Yes

Are the conclusions drawn adequately supported by the results?

Yes

Are sufficient details of methods and analysis provided to allow replication by others?

Yes

Reviewer Expertise:

Tourism, Hospitality, PLS-SEM

I confirm that I have read this submission and believe that I have an appropriate level of expertise to confirm that it is of an acceptable scientific standard.
